# Understanding the measurement relationship between EQ-5D-5L, PROMIS-29 and PROPr

**DOI:** 10.1007/s11136-023-03462-6

**Published:** 2023-06-22

**Authors:** Brendan J. Mulhern, Tianxin Pan, Richard Norman, An Tran-Duy, Janel Hanmer, Rosalie Viney, Nancy J. Devlin

**Affiliations:** 1 Centre for Health Economics Research and Evaluation, University of Technology Sydney, Ultimo, Australia; 2 Health Economics Unit, Centre for Health Policy, Melbourne School of Population and Global Health, University of Melbourne, Parkville, Australia; 3 School of Population Health, Curtin University, Perth, Australia; 4 Department of General Internal Medicine, University of Pittsburgh, Pittsburgh, PA USA

**Keywords:** EQ-5D-5L, PROMIS, Health-related quality of life, Psychometrics

## Abstract

**Purpose:**

Many generic patient-reported instruments are available for the measurement of health outcomes, including EQ-5D-5L, and the Patient-Reported Outcome Measurement Information System (PROMIS). Assessing their measurement characteristics informs users about the consistency between, and limits of, evidence produced. The aim was to assess the measurement relationship between the EQ-5D-5L descriptive system and value sets, the PROMIS-29 and PROPr (PROMIS value set).

**Methods:**

Data were extracted from a cross-sectional survey administering measures of quality of life online in Australia. Descriptive analysis, agreement and construct validity assessment methods were used to compare instruments at the item, domain and value set level.

**Results:**

In total, 794 Australians completed the survey. Convergent validity analysis found that similar dimensions across instruments were highly correlated (> 0.50), but the PROMIS-29 assesses additional health concepts not explicitly covered by EQ-5D (sleep and fatigue). Known-group assessment found that EQ-5D-5L and PROPr were able to detect those with and without a condition (ES range 0.78–0.83) but PROPr could more precisely detect differing levels of self-reported health. Both instruments were sensitive to differences in levels of pain.

**Discussion:**

There is some consistency in what the EQ-5D-5L, PROMIS-29 and PROPr measure. Differences between value set characteristics can be linked to differences what is measured and the valuation approaches used. This has implications for the use of each in assessing health outcomes, and the results can inform decisions about which instrument should be used in which context.

**Supplementary Information:**

The online version contains supplementary material available at 10.1007/s11136-023-03462-6.

## Plain English Summary

Health-related quality of life information is used in clinical decision-making and also supports the allocation of funds in the health care system. There are many different questionnaires used to measure health-related quality of life, but we do not know which questionnaire is most appropriate in different groups of people. In this study, we compare three popular ways of measuring health-related quality of life to understand the relationship between them. The results of the study suggest that the concepts of quality of life measured by the questionnaires have both similarities and differences. And the scoring systems have different characteristics that could lead to different inputs into clinical decision-making and about how to fund the healthcare system.

## Introduction

There is a range of generic and condition-specific patient-reported instruments available for the measurement of health-related quality of life (HRQoL).^1^ Generic instruments play an important role in providing evidence that can be compared across disease areas and populations, as it is required to inform decisions affecting resource allocation. They can be used to assess the HRQoL associated with a condition or population, understand change over time and to inform clinical decision-making.

Some generic instruments are accompanied by value sets which enable the data generated by the instruments to be summarised in a manner that reflects preferences of a population (e.g. the general public from different countries) about the relative importance of the domains/dimensions. Value sets are generated using a preference elicitation technique such as the time trade off (TTO) or discrete choice experiments (DCE) [1]. These methods generate preference data for a subset of health states described by an instrument, and the data are modelled to estimate a value for every health state described by an instrument (known as the utility value). This results in an overall value set which is anchored on a scale from full health = 1 to dead = 0, and is used in the estimation of quality-adjusted life years (QALYs) to inform the economic evaluation of health care. QALYs are calculated by multiplying the time spent in a particular health state by the utility value of that health state. Therefore, a year in full health is equivalent to 1 QALY, and death has 0 QALYs.

There are a number of generic instruments for which value sets are available, such as the EQ-5D-3L [2] and EQ-5D-5L [3]). The EQ-5D is the most widely used generic measure internationally, and its evidence is used in a wide range of contexts including resource allocation decision-making [4–6], routine outcome measurement, clinical trials and population health surveys. There are over 25 EQ-5D-5L value sets available internationally [7] that reflect the health preferences of the population of each particular country. For a full description of the EQ-5D-5L, see Table 1.

**Table 1:** Description of the EQ-5D-5L, PROMIS-29 and PROPr

Instrument	General structure	Dimensions/domains measured	Response levels	General scoring description	Value set description
EQ-5D-5L	Five single item dimensions	Mobility (MO) Self-Care (SC) Usual Activities (UA) Pain/Discomfort (PD) Anxiety/Depression (AD)	Five response levels framed as ‘problems’ (No, Slight, Moderate, Severe, Extreme problems/Unable to)	Scored using value sets based on population preferences derived using a preference elicitation technique	Australian value set: Ranges from 1 to -0.676 US value set: Ranges from 1 to − 0.573
PROMIS-29 (29 items, 7	29 items measuring seven domains (4 items each), and a single pain intensity numeric rating scale	Depression (D) Anxiety (A) Physical Function (PF) Pain Interference (PA) Fatigue (FA) Sleep Disturbance (SL) Ability to Participate in Social Roles and Activities (SOC)	Five response levels for 28 items included in domains Different response categories across domains: PF: Without any difficulty–Unable to do A, D, SOC: Never–Always F, PA, SL (3 items): Not at all–Very much SL (1 item): Very poor–Very good Pain intensity item scored on 0–10 scale	Domain raw scores (from 4–20) are converted into T-scores, which have a mean of 50 and a standard deviation of 10. High scores represent more of the trait being measured	N/A
PROPR	Estimated from seven PROMIS domains	PF D F PA SL SOC Cognitive Function (CF)	N/A	Calculated from the PROMIS-29 using a mapping function to predict the missing CF score based on the domain scores included in PROMIS-29. Each domain T-score is mapped to its own domain utility score and then these are combined into a utility score	PROPr values based on the PROMIS-29 range from 0.954 to –0.022

Recently, the Patient-Reported Outcomes Measurement Information System (PROMIS) initiative has developed calibrated item banks for generic health domains using Item Response Theory (IRT) [8, 9]. PROMIS measures can be administered as standardised short forms, via computer adaptive testing, or in a custom form. The PROMIS-29 (described in Table 1) [10] is a fixed-form profile measure adapted from a reduced set of PROMIS item banks.

The PROMIS-Preference scoring system (PROPr, also described in Table 1) [11] is based on seven PROMIS domains, and PROPr utilities based on the PROMIS-29 can be derived [12]. Preferences for PROMIS health domains were elicited using the standard gamble (SG) approach. Currently, only a value set based on US general population preferences has been developed.

The PROMIS item banks and PROMIS-29 are becoming established in many health settings in the US and are being promoted internationally (for example, see Evans et al. [13]). The PROMIS-29 is the most widely used PROMIS profile measure [12]. The EQ-5D is in established use in many countries. The proliferation of instruments within the same measurement space, but developed using different approaches, means that an assessment of the measurement characteristics of each measure is required. This allows for an understanding of the usefulness of each instrument in different populations and informs users about the consistency between evidence produced by each.

A recent review of studies comparing the measurement properties of the EQ-5D and PROMIS-29 [14] found six that focused on the relationship between the EQ-5D-5L and PROMIS-29 [15–20]. A number of these focused on construct validity, finding evidence supporting the known-group validity of both measures across health condition groups [15, 16], and also mixed evidence of convergence between the EQ-5D-5L dimensions or values, and PROMIS-29 domains, where expected [17, 18]. The strength of evidence supporting instrument responsiveness at the dimension or domain level also varied [19, 20].

Subsequent to the review, a comparative analysis of PROPr utilities and EQ-5D-5L value sets based on the theoretical values found clear differences in value set properties [21]. This included different value set ranges meaning that PROPr has lower values for comparable mild health states, and higher values for severe states. The importance of dimensions also differs, with pain having a larger relative utility decrement for EQ-5D-5L than for PROPr. Rencz and colleagues [22] found good convergent validity between EQ-5D-5L and PROMIS-29 domains capturing similar aspects of health. The diversity of findings highlights the importance of further comparisons of the instruments using patient-reported data. Examining further how each instrument performs in different health areas advances knowledge about their measurement characteristics.

Therefore, the aim of this study was to assess the measurement relationship between the EQ-5D-5L, PROMIS-29 and PROPr using self-reported data from Australia. This was done using the tests of agreement and construct validity to generate the evidence to understand the use of EQ-5D-5L and PROMIS-29 as alternative measures of patient-reported outcomes.

## Methods

### Data and sample

The data used in this study were extracted from a survey administering measures of HRQoL online to patients with common conditions and the general population, in Australia [23]. The common conditions targeted included diabetes, depression, pain and arthritis. Respondents were invited via email and online advertisements. The instruments in the survey were completed in a random order. Therefore, approximately half of the sample completed the EQ-5D-5L prior to the PROMIS-29 and vice versa. Demographic and self-reported health questions were also administered. The survey is described in more detail in Mulhern [23].

### Measures and value sets

#### EQ-5D-5L

As described in Table 1, the EQ-5D-5L measures health on five dimensions with five response levels. Multiple EQ-5D-5L value sets were used for the comparisons conducted in this study. These included the pilot Australian value set based on a DCE [24] and the United States value set [25] that used the EQ-VT protocol (combining TTO and DCE) [1]. The Australian value set was used due to the inclusion of Australian respondents. The US value set was used for direct comparisons with the PROPr value set as both are based on the preferences of the US population. As described in Table 1, these differ in terms of the overall value set range, where the value for the worst health state with extreme problems on each dimension is lower (i.e. valued as poorer) for the Australian population than for the US population.

#### PROMIS-29

PROMIS-29 is the shortest of the PROMIS Profile measures (see Table 1) and was included in the survey to generate Australian evidence about its psychometric properties and its measurement relationship with other generic HRQoL instruments. The raw scores for each domain were converted into T-scores based on the look up tables in the PROMIS scoring manual [26]. High scores represent more of the trait being measured, so a high score on PF and SOC indicates good functioning in both domains. A high score on the other dimensions indicates a higher level of problems.

#### PROPr

PROPr was developed in the US [11] and allows preference-based scores to be estimated from health states described by 7 PROMIS domains (see Table 1 for a description) and can be estimated from the PROMIS-29 [18]. The PROMIS-29 and PROPr share six of these domains (PF, D, F, PA, SL, SOC). The final domain (CF) domain is not included in PROMIS-29, so it had to be imputed. The currently recommended approach is to collect PROMIS-29 + 2 v2.1. However, this was not possible in this study, as the data collected predated the recommendation, so the imputation approach developed by Dewitt and colleagues [12] that estimates CF using linear regression approaches was used.

### Data analysis

Descriptive analysis, agreement and construct validity assessment methods were used to compare the instruments and value sets at the item, dimension, domain and value set level.

#### Descriptive comparisons of the items, dimensions and value sets

EQ-5D-5L and PROPr utilities, and PROMIS-29 domain T-scores were compared descriptively. The internal consistency of the PROMIS-29 domain scores was estimated using Cronbach’s Alpha (where a range of 0.70–0.95 was used to indicate a positive rating of internal consistency [27]). The overall distribution of utility values was displayed using histograms, and we used Pearson’s moment coefficient of skewness to compare distributions (a coefficient of 0 is normal, 1 half-normal and 2 exponential). The frequencies of commonly reported EQ-5D-5L health states and PROMIS-29T-score patterns were also examined.

#### Agreement between value sets

We compared the agreement between the EQ-5D-5L and PROPr value sets using Bland–Altman plots. These present the mean of two scores on the *x*-axis and the difference on the *y*-axis, with lines indicating the upper and lower limits of agreement [calculated as the mean difference ± 1.96 × standard deviation (SD)] added. Any responses outside of these limits indicate disagreement between the responses to each measure.

#### Construct validity—convergence

Convergent validity is a form of construct validity, and assesses whether instruments measure similar or different constructs (in the absence of a ‘gold standard’ measure of HRQoL). We summarised the relationship between the EQ-5D-5L dimensions and PROMIS-29 domains, EQ-5D-5L dimensions and PROPr utility values, and PROMIS-29 domains and EQ-5D-5L values using Spearman correlation coefficients. Correlations were conducted not only for the overall sample, but also for the subgroups of those reporting physical (including back pain, hypertension, breathing problems, diabetes, arthritis and heart disease) and mental health (including anxiety and depression) conditions. Correlations of above 0.5 were considered strong [28].

#### Construct validity—known-group differences

Known-group validity is also a form of construct validity and assesses the sensitivity of instruments to detect the differences between samples with different characteristics where responses might be expected to differ. We compared the known-group validity across instruments for a number of subgroups including the presence or absence of any health condition, overall physical and mental health conditions, health problems reported by more than 20% of the sample (pain, tiredness, anxiety, depression, hypertension), the number of comorbid health conditions (0, 1–2 and 3 or more), self-reported health and health satisfaction (based on responses to the 10-point question, where low health satisfaction was defined as a score of zero to five, and high a score of six or more). We also assessed known-group validity based on health service use indicators including visits to a general practitioner (GP), and overnight hospitalisations, in the previous year. The magnitude of the difference was assessed using Cohen’s *d* effect size and one-way ANOVA group difference testing. Cohen’s *d* effect size is a standardised measure of group differences calculated using Eq. 1, where *M* is the mean score of each group, and the pooled standard deviation (*σ*_pooled_) is calculated using Eq. 2:

1 $$ {\text{Cohen's}} \;d = (M_{{1}} - M_{{2}} ) /\sigma_{{{\text{pooled}}}} , $$

2 $$ \sigma_{{{\text{pooled}}}} = \, \surd \, [(\sigma_{1}^{2} + \sigma_{2}^{2} )/2]. $$

Effect sizes are benchmarked as small (*d* = 0.2), medium (*d* = 0.5) and large (*d* = 0.8) [28].

## Results

### Data and sample

In total, 794 respondents (87.5% of those accessing the survey) fully completed the survey. Of the 113 (12.5%) who accessed the survey but did not fully complete, 40 (4.4%) dropped out prior to completing any question, leaving 73 (8.0%) answering at least one survey question. Of these, 49 (5.4%) did not complete any EQ-5D-5L or PROMIS-29 questions, meaning 24 (2.6%) provided incomplete EQ-5D-5L or PROMIS-29 data (and therefore, sensitivity analysis including this small number of respondents was not conducted.) The mean time to complete the survey was 29 min (range 4.5–174.4 min). Table 2 reports sample demographics. Overall, 500 (63%) of the sample reported having at least one long-term health condition, with 52% reporting comorbid health conditions.

**Table 2:** Sample demographics

Category	N (%)
*Overall*	794
*Age*	
18–29	128 (16.1)
30–44	202 (25.4)
45–59	222 (28.0)
60–74	220 (27.7)
75+	20 (2.5)
*Gender*	
Male	380 (47.9)
Female	414 (52.1)
*Country of birth*	
Australia	623 (78.9)
Other	167 (21.1)
*Health Conditions*	
Pain	228 (28.8)
Tiredness	217 (27.4)
Depression	195 (24.6)
Anxiety	169 (21.3)
High blood pressure	166 (21.0)
Insomnia	111 (14.0)
Breathing problems	110 (13.9)
Diabetes	107 (13.5)
Arthritis	104 (13.1)
Heart disease	40 (5.1)
Cancer	19 (2.4)
Stroke	10 (1.3)
*Number of conditions*	
0	292 (36.8)
1	93 (11.7)
2	119 (15.0)
3 +	288 (36.3)
*Visits to GP in last year*	
0	62 (7.8)
1–2	201 (25.3)
3–5	250 (31.5)
6+	281 (35.3)
*Income*	
0 to 80,000 AUD	585 (73.7)
80,001 AUD plus	134 (16.9)
Prefer not to say	75 (9.5)
*Marital status*	
Married/de facto	465 (58.6)
Separated/divorced/single/widowed	329 (41.4)
*Have children*	389 (49.0)
*Education level*	
Bachelors/higher degree	280 (35.3)
Trade certificate/diploma	247 (31.1)
Primary/secondary	267 (33.6)

### Descriptive analysis and comparisons of the items, dimensions and value sets

Table 3 reports the descriptive statistics for the EQ-5D-5L, PROMIS-29 and PROPr. The utilities for PROPr differ from those for the EQ-5D-5L, both in terms of the mean value, which is substantially lower, and the smaller range of values reported. The EQ-5D-5L scores also differ significantly across the value sets, particularly in terms of the range of values for the same health states. The best health state value on PROPr is 0.905 which does not equate to full health. The mean PROMIS-29 domain T-scores are between 47 and 53 (with SDs between 8.6 and 10.2) indicating a level of equivalence with the US population. The internal consistency of the PROMIS-29 domains ranges from 0.86 to 0.95 and is therefore positively rated.

**Table 3:** Descriptive statistics of the EQ-5D-5L and PROMIS-29 (utility and T-scores)

Measure and dimension	Mean (SD)	Median	Range	Alpha
*PROMIS-29*				
Depression	53.3 (10.1)	53.9	41.0 to 79.4	0.95
Anxiety	53.7 (9.8)	53.7	40.3 to 81.6	0.92
Physical functioning	47.3 (8.6)	48.3	22.5 to 57	0.92
Pain	52.6 (9.2)	53.9	41.6 to 75.6	0.95
Fatigue	53.0 (10.2)	51.0	33.7 to 75.8	0.95
Sleep	52.0 (9.0)	52.4	32.0 to 73.3	0.86
Social limitations	51.4 (9.2)	51.9	27.5 to 64.2	0.93
PROPr utility	0.405 (0.232)	0.388	− 0.021 to 0.905	
*EQ-5D utility*				
Australian	0.699 (0.276)	0.754	− 0.676 to 1	
United States	0.759 (0.251)	0.844	− 0.573 to 1	

There is evidence of a ceiling effect for EQ-5D-5L at the item (Appendix 2) and overall level (Appendix 3), where 17.5% of the sample report they are in the best health state, whereas there is no ceiling effect for PROMIS-29 (Appendix 3 and 4). Overall, the sample report 192 unique EQ-5D-5L health states (6.1% of all possible). Only the top five most common states are reported more than 20 times, with 117 of the 192 reported only once. Comparing this to PROMIS-29, 14 (1.8%) respondents report themselves to be in the best possible profile. Of these 14, 12 are also in the best EQ-5D-5L health state.

**Fig. 1:**
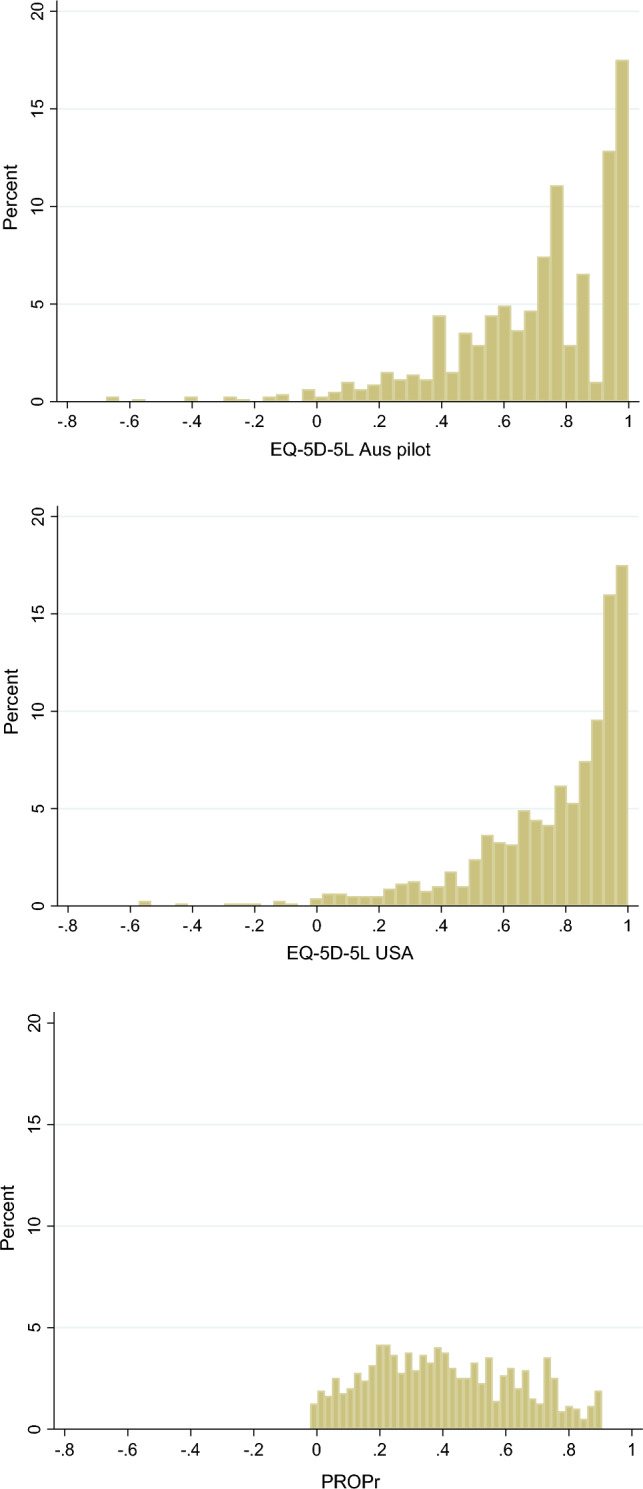
Distribution of value-weighted data

Figure 1 displays histograms of the overall value set distributions, where differences between the EQ-5D-5L and PROPr are observed. The EQ-5D-5L utilities display a peak of values for mild and moderate problems and a tail with a limited number of negative values. The PROPr values are more evenly distributed across the positive range of the utility scale. Pearson’s moment coefficient of skew indicated that the Australian and US value sets were less normal and exhibited skew in the opposite direction than PROPr (− 1.34, − 1.69 and 0.23, respectively, where a score closer to zero indicates a more normal distribution).

### Agreement between value sets

**Fig. 2:**
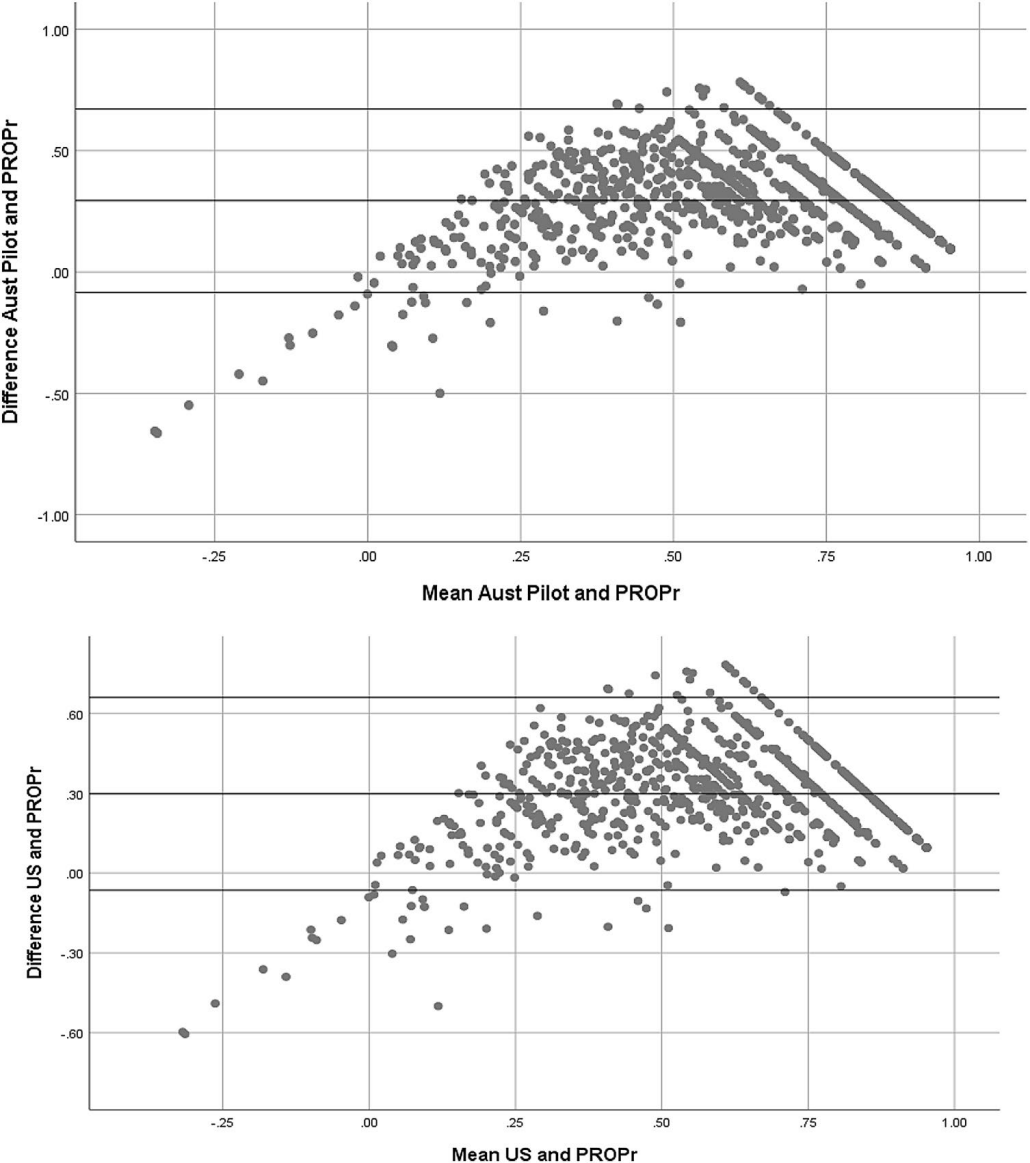
Bland Altman plots showing agreement between value sets

Figure 2 reports the Bland Altman agreement plots between the EQ-5D-5L value sets and PROPr. The results indicate a generally good level of agreement across the utility range, with limited disagreement when respondents indicate higher impairment (i.e. have a low mean score across the two instruments).

### Convergent validity

Table 4 reports the correlations between dimensions and value sets as an indicator of convergence for the overall sample and for those with physical and mental health conditions. Appendices 5 (EQ-5D-5L) and 6 (PROMIS-29) include within instrument dimension and domain correlations as a comparison. Correlations in bold are in the range defined as strong (> 0.5). At the dimension level, strong correlations are found between dimensions where the concepts measured were hypothesised to be similar (EQMO and PROMIS PF, EQ UA and PROMIS SOC, EQ PD and PROMIS PA, EQ AD and PROMIS A and D). There is also evidence of a strong relationship between other dimensions (including EQ MO and PROMIS PA and SOC, EQ SC and PROMIS PF, EQ PD and PROMIS SOC, and EQ AD and PROMIS F, SL and SOC). These results demonstrate the overlap between HRQoL constructs measured. The lower correlations with PROMIS F and SL suggests that fatigue and sleep problems are indirectly, but not explicitly, measured by EQ-5D-5L.

**Table 4:** Convergent validity of EQ-5D-5L dimensions, utilities, PROMIS-29 domain and PROPr utilities

	EQ-5D-5L dimensions					EQ-5D-5L value sets	
	Mobility	Self-care	Usual activities	Pain/discomfort	Anxiety/depression	Australian	United States
	*Whole sample*						
PROMIS dimension							
Depression	0.29	0.29	0.45	0.35	*0.75*	**− 0.64**	**− 0.58**
Anxiety	0.29	0.32	0.44	0.32	*0.73*	**− 0.63**	**− 0.56**
Physical functioning	*− 0.76*	**− 0.51**	*− 0.69*	− 0.57	− 0.28	**0.66**	**0.72**
Pain	**0.61**	0.46	*0.65*	*0.71*	0.35	**− 0.70**	**− 0.74**
Fatigue	0.32	0.22	0.45	0.43	**0.50**	**− 0.56**	**− 0.52**
Sleep	0.30	0.20	0.40	0.41	**0.50**	**− 0.53**	**− 0.50**
Social limitations	**− 0.54**	− 0.37	*− 0.65*	**− 0.54**	**− 0.52**	**0.71**	**0.69**
PROPr utility	**− 0.52**	− 0.36	**− 0.63**	**− 0.58**	**− 0.59**	**0.74**	**0.71**
	*Physical health condition (n = 443)*						
PROMIS dimension							
Depression	0.28	0.33	0.47	0.36	*0.76*	**− 0.67**	**− 0.60**
Anxiety	0.26	0.34	0.43	0.34	*0.74*	**− 0.64**	**− 0.58**
Physical functioning	*− 0.79*	**− 0.52**	*− 0.72*	**− 0.55**	− 0.21	**0.67**	**0.72**
Pain	**0.60**	0.46	*0.68*	*0.74*	0.30	**− 0.70**	**− 0.75**
Fatigue	0.30	0.25	0.45	0.39	0.46	**− 0.54**	**− 0.50**
Sleep	0.28	0.20	0.40	0.40	0.49	**− 0.52**	− 0.49
Social limitations	**− 0.54**	− 0.39	*− 0.67*	**− 0.52**	− 0.45	**0.69**	**0.69**
PROPr utility	**− 0.51**	− 0.37	**− 0.64**	**− 0.58**	**− 0.55**	**0.73**	**0.71**
	*Mental health condition (n = 243)*						
PROMIS dimension							
Depression	0.22	0.23	0.42	0.26	*0.69*	**− 0.56**	**− 0.52**
Anxiety	0.21	0.27	0.39	0.17	*0.63*	**− 0.51**	− 0.47
Physical functioning	*− 0.76*	− 0.50	*− 0.66*	**− 0.57**	− 0.21	**0.67**	**0.72**
Pain	**0.60**	0.39	*0.62*	*0.74*	0.21	**− 0.67**	**− 0.71**
Fatigue	0.16	0.13	0.35	0.33	0.36	− 0.40	− 0.38
Sleep	0.13	0.07	0.30	0.27	0.41	− 0.39	− 0.37
Social limitations	− 0.45	− 0.34	*− 0.63*	− 0.44	− 0.43	**0.63**	**0.63**
PROPr utility	− 0.42	− 0.29	**− 0.60**	**− 0.51**	**− 0.51**	**0.66**	**0.65**

Italicised cells indicate hypothesised relationship between dimensions; Correlations in bold are in the range defined as strong (0.5+)

Regarding within instrument relationships, there is evidence of strong correlations between EQ MO and EQ SC, EQ UA and EQ PD, and EQ UA with EQ SC and EQ PD. EQ AD is not highly correlated with any other dimension (Appendix 5). PROMIS A and D are strongly correlated with each other and also F, SL and SOC (which is strongly correlated with all other domains). PA, F and PF are also strongly correlated (Appendix 6).

At the dimension and value set level, the PROMIS dimensions are strongly correlated with the EQ-5D-5L value sets at a generally higher level than the EQ-5D-5L dimensions are with PROPr. There is a low correlation between EQ SC and PROPr. The correlations between the EQ-5D-5L value sets and PROPr are strong. This demonstrates that at the utility level, there is a strong relationship between the values. However, some of the differences in the measurement relationship between dimensions are not detected at the utility level.

The correlation patterns described above are consistent across those with a physical and mental health conditions. One key difference is that the correlations for those with a mental health condition are almost consistently lower than those with a physical health condition. This suggests some divergence in the relationship between the instruments in different condition groups.

### Known-group validity

Table 5 reports the known-group validity indicators. The results suggest that both the EQ-5D value sets and PROPr can distinguish between the majority of groups, indicated by the effect sizes in the high range. The lowest level of discriminance for both instruments is for distinguishing between number of GP visits (ES range 0.12–0.53). PROPr distinguished between levels of self-reported health at a higher level than EQ-5D-5L indicating PROPr might be more sensitive in the general population and patients with mild problems. EQ-5D-5L and PROPr distinguish between the groups defined by the presence or absence of a health condition (EQ range 0.78–0.83). The EQ-5D has a higher, but small effect size difference between groups defined as having zero conditions and one to two conditions [ES 0.42/0.45 (EQ) vs 0.24 (PROPr)], but PROPr displays higher differences between those with one of two conditions, and those with three or more [ES 0.69/0.86 (EQ) vs 1.10 (PROPr)]. All instruments are sensitive to Physical Health (EQ range 0.76–0.80) and mental health conditions (ES range 0.98–1.17) in similar ranges, but more sensitive to mental health impacts. PROPr is more sensitive to differences in health satisfaction [ES 0.79/0.78 (EQ) vs 0.99 (PROPr)]. Appendix 7 reports the validity statistics across the top five most reported health conditions. All three value sets are sensitive to pain, depression and anxiety differences at a strong level (ES range 0.98–1.21), but less sensitive to the impacts of hypertension (ES range 0.38–0.50). PROPr is more sensitive to tiredness concerns than both EQ-5D-5L value sets [ES 0.76/0.68 (EQ) vs 1.07 (PROPr)].

**Table 5:** Known-group validity across the value sets

	*N*	EQ-5D Australian			EQ-5D-5L United States			PROPr		
		Mean (SD)	ES (95% CI)	Sig	Mean (SD)	ES (95% CI)	Sig	Mean (SD)	ES (96% CI)	Sig
*Condition*			**0.83 (0.68–0.98**	< 0.001		**0.80 (0.65–0.95)**	< 0.001		0.78 (0.63–0.93)	< 0.001
No	292	0.834 (0.182)			0.879 (0.147)			0.513 (0.224)		
Yes	500	0.623 (0.289)			0.692 (0.271)			0.343 (0.213)		
*Multimorbidity*				< 0.001			< 0.001			< 0.001
No condition	292	0.834 (0.182)	0.42 (0.24–0.60)		0.879 (0.147)	0.45 (0.27–0.63)		0.513 (0.224)	0.24 (0.06–0.42)	
One–two conditions	212	0.741 (0.264)	0.86 (0.67–1.04)		0.792 (0.242)	0.69 (0.50–0.87)		0.461 (0.209)	**1.10 (0.91–1.29)**	
Three or more conditions	290	0.533 (0.278)			0.615 (0.269)			0.255 (0.170)		
*Physical health condition*			0.78 (0.63–0.92)	< 0.001		**0.80 (0.65–0.94)**	< 0.001		0.76 (0.61–0.90)	< 0.001
No	351	0.811 (0.207)			0.863 (0.171)			0.497 (0.224)		
Yes	443	0.611 (0.291)			0.678 (0.273)			0.332 (0.212)		
*Mental health condition*			**1.17 (1.01–1.33)**	< 0.001		**0.98 (0.82–1.14)**	< 0.001		**1.12 (0.96–1.28)**	< 0.001
No	551	0.786 (0.224)			0.828 (0.205)			0.476 (0.221)		
Yes	243	0.502 (0.281)			0.604 (0.276)			0.245 (0.168)		
*Self-rated health*				< 0.001			< 0.001			< 0.001
Poor-fair	226	0.523 (0.307)	0.77 (0.59–0.95)		0.601 (0.298)	0.64 (0.47–0.82)		0.232 (0.167)	**0.95 (0.77–1.13)**	
Good	297	0.728 (0.227)	0.38 (0.22–0.55)		0.760 (0.200)	0.50 (0.34–0.67)		0.409 (0.199)	0.65 (0.48–0.82)	
Very good–excellent	271	0.814 (0.220)			0.858 (0.189)			0.545 (0.217)		
*GP visits*				< 0.001			< 0.001			< 0.001
0	62	0.809 (0.175)	0.12 (− 0.16–0.12)		0.864 (0.136)	0.13 (0.15–0.42)		0.481 (0.233)	0.02 (− 0.30–0.27)	
1 to 2	201	0.781 (0.243)	0.25 (0.06–0.43)		0.838 (0.214)	0.27 (0.08–0.45)		0.485 (0.234)	0.26 (0.07–0.44)	
3 to 5	250	0.721 (0.245)	0.44 (0.27–0.61)		0.780 (0.220)	0.45 (0.28–0.62)		**0.426 (0.227)**	0.53 (0.36–0.70)	
6 +	281	0.597 (0.308)			0.663 (0.288)			0.312 (0.203)		
*Health satisfaction*			0.79 (0.85–0.95)	< 0.001		0.78 (0.62–0.94)	< 0.001		**0.99 (0.83–1.15)**	< 0.001
Low	223	0.551 (0.302)			0.626 (0.290)			0.254 (0.190)		
High	571	0.757 (0.241)			0.812 (0.213)			0.464 (0.220)		
*Hospitalised overnight*			0.65 (0.48–0.82)	< 0.001		0.67 (0.50–0.84)	< 0.001		0.57 (0.40–0.74)	< 0.001
No	613	0.739 (0.245)			0.797 (0.220)			0.434 (0.228)		
Yes	181	0.566 (0.328)			0.635 (0.305)			0.305 (0.217)		

Large effect sizes (< 0.8) highlighted in bold

## Discussion

Comparisons between generic HRQoL instruments are important to help understand how any differences may impact on the evidence generated to support clinical and health care decision-making. This study has added to the sparse literature comparing the EQ-5D-5L and PROMIS-29 descriptive systems, and EQ-5D-5L value sets and PROPr. The results build on earlier work comparing the EQ-5D and PROMIS-29-based instruments by Pan et al. [14] and support emerging evidence of an interaction between the measurement and valuation properties of the instruments.

At the descriptive system level, both measures exhibit acceptable properties, particularly in detecting differences between the self-reported health levels of respondents. There is evidence of consistency between dimensions measuring similar constructs; however, each measure also includes constructs not explicitly assessed by the other. Response patterns also differ somewhat, with more respondents likely to report no problems on EQ-5D-5L dimensions compared to PROMIS domains. This is expected given each PROMIS-29 domain includes four items rather than one and asks questions in different way, using a combination of frequency and severity (see Table 1). Differences in how HRQoL is measured might also be due to the methods used to develop the instruments. For example, the use of IRT for the development of PROMIS ensures that the items were psychometrically validated from the initial development phase, and the selection of the items for the short form PROMIS-29 was psychometrically supported from a longer item bank. The development of the EQ-5D was less psychometrics focused; however, many studies have demonstrated its psychometric validity and limitations across conditions [29].

There are implications of these measurement differences for the choice of profile measures between the EQ-5D-5L and PROMIS-29 in clinical settings and decision-making. PROMIS-29 provides a more extensive profile of HRQoL that can be compared to other PROMIS item banks and fixed forms as well as other HRQoL instruments. PROMIS also results in individual domain level scores which provide an additional level of patient-reported information. The lower correlations between EQ SC and PROMIS domains may suggest that self-care is not clearly captured by the PROMIS-29. However, this is confounded by the low variation in SC scores displayed by the sample (see Appendix 2), and further research could examine this issue in a patient population with a higher level of self-care-related issues. Similarly, the EQ-5D-5L could be limited in populations where sleep problems and fatigue are important concerns. It is also insightful to compare how the items within dimensions might drive the relationships observed. For example, PROMIS PA is highly correlated with the MO, UA and PD dimensions, but not SC. This may be explained by the questions included in PA which ask about pain interference in day-to-day activities, work around the home, ability to participate in social activities and household chores.

A recent measurement characteristic of EQ-5D-5L that has received attention in the literature is the use of ‘composite’ dimensions that measure two constructs (PD and AD) [30–32]. The correlations between EQ PD and PROMIS PA, and EQ AD and PROMIS A and D inform this issue to some extent. The strong correlation between the pain items suggests that pain is measured by both, but we do not have information to understand the extent to which discomfort is considered. That both PROMIS A and D have a strong correlation with EQ AD suggests that both are considered, but the composite response does not allow for detailed understanding of which concept is being referred to. Measuring anxiety and depression separately is a benefit of a longer profile measure such as PROMIS. Further analysis could examine the measurement relationship between EQ AD and each of the eight items included in the PROMIS-29That measure anxiety and depression.

Regarding value set comparisons, there is a variable relationship between the instrument dimensions and domains and value sets, and between value sets. Taking the former, the strong relationship between the PROMIS-29 dimensions and EQ-5D-5L value sets indicates that the areas of HRQoL measured by the PROMIS-29 are reflected in EQ-5D utilities, even if some are not explicitly measured in the descriptive system such as fatigue. This is also in line with a US study that mapped five PROMIS domain T-scores (PF, F, PA, A and D) to EQ-5D-3L utility [33] and found that fatigue is important in EQ-5D-3L utilities. The lower correlation between PROPr and the EQ-5D-5L dimensions suggests less of an overlap, particularly for EQ SC. This could be a result of the value set development approaches, where EQ-5D-5L values were estimated from full EQ-5D health state descriptions, but the development of PROPr focused on valuing corner states, with one health issue described at a time.

In comparing value sets, at the overall level there is evidence of a strong relationship and level of agreement, and all of the value sets exhibit strong known-group validity. However, there is evidence that the different value set characteristics [14] exert an effect on the distributions of data evident in this sample. The strong overall correlation masks the measurement differences highlighted previously at the utility level. The value set characteristics also differ due to the methods used to develop the value sets. TTO and SG differ in their approach to eliciting values, and the states selected for valuation also impact the models produced (a consequence of valuing two measures with contrasting approaches to measuring health). Further work could compare the values produced for each measure using the same valuation approach.

Considering individual conditions and impacts on health, it is evident that both EQ-5D-5L and PROPr are sensitive to differences in heath concepts that are directly assessed by the instruments (including pain and mental health). PROPr is more sensitive to issues around tiredness given similar concepts are assessed by the PROMIS-29. The instruments do not detect differences between those with and without high blood pressure. This is not unexpected, as high blood pressure is generally asymptomatic, and well controlled following diagnosis, and therefore any HRQoL impacts may not be detected by the instruments. In related work, Hanmer [34] found that PROPr associates with social determinants of health at a higher level than EQ-5D-5L, and both instruments are sensitive to issues around food security [35]. This adds to the complex picture of where and in what populations instruments should be used, and further work could extend the analysis to include other health conditions and social impacts on health.

There are implications of the value set characteristics for the use of both in QALY estimations in decision-making process. For example, the value given to the best health state varies (1 for EQ-5D-5L and 0.905 for PROPr based on PROMIS-29). The PROPr utilities range is smaller indicating that, even though PROMIS-29 produces more possible health states, large change in health as measured by the PROMIS-29 may not be reflected in PROPr to the same extent that a matched descriptive change would be reflected in an EQ-5D-5L value set. Longitudinal patient and/or clinical trial data including both the EQ-5D-5L and PROMIS-29 are required to explore this.

There are a number of limitations with this study that have to be taken into account when considering the generalisability of the results. First, the data were collected online, and therefore, we did not have control over the environment in which it was collected. Online self-report surveys are now more widely used and accepted for the collection of outcomes data and have been successfully collected in Australia previously [36]. Another issue with the use of online panel respondents to assess the measurement relationships between instruments is that the sample reports generally mild health impairments, so generalising the results of the comparison to more severe health problems requires careful consideration. We also focused on a comparison of health-related QoL measures. Recently, measures of QoL using different perspectives have been developed. For example, the Adult Social Care Outcomes Toolkit (ASCOT) [37] measuring social care QoL, and the EuroQol-Health and Wellbeing (EQ-HWB) [38] measuring aspects of broader QoL relating to both health and social care. Further work needs to understand the relationship between a broader range of outcome measures. A final limitation relates to the use of imputation to estimate PROPr CF values. This was done as only the PROMIS-29 was included in the survey. However, in the development of the imputation methods, the level of error in the estimates was small [12]. This provides a basis for supporting the validity of the estimates used in this study.

In conclusion, we have demonstrated that the strength of the measurement relationship between the EQ-5D-5L, PROMIS-29 and PROPr differs depending on which validity indicator is used and also differs depending on whether the items and dimensions, or value sets, are compared. This has implications for the use of each in the assessment of health, and subsequent decision-making as outlined above. The development of the PROMIS system and availability of PROPr have potential implications for the use of EQ-5D internationally. Further work to understand the advantages and disadvantages of each in different populations is warranted.

### Supplementary Information

Below is the link to the electronic supplementary material.

Supplementary file1 (DOCX 41 KB)

## Data Availability

The data used in this study is available on request from the corresponding author via a data sharing agreement process.

## Footnotes

1 Appendix 1 includes a list of abbreviations used in this paper.
