# Supplementary material for: Understanding the measurement relationship between EQ-5D-5L, PROMIS-29 and PROPr
Source: Qual Life Res. 2023 Jun 22;32(11):3147–60. doi: 10.1007/s11136-023-03462-6 (PMC10522725; doi:10.1007/s11136-023-03462-6)
Supplement: Supplementary file 1 — Supplementary file1 (DOCX 41 KB) [file 11136_2023_3462_MOESM1_ESM.docx]

Supplementary material for Mulhern B, Pan T, Norman R, Hanmer J, Viney R, Devlin N. Understanding the measurement relationship between EQ-5D-5L, PROMIS-29 and PROPr

**Appendix 1: Table of abbreviations:**

| **Abbreviation** | **Meaning** |
| --- | --- |
| A | Anxiety (PROMIS-29 and PROPR dimension) |
| AD | Anxiety and Depression (EQ-5D dimension) |
| ANOVA | Analysis Of Variance |
| ASCOT | Adult Social Outcomes Toolkit |
| CF | Cognitive Functioning (PROPR dimension) |
| D | Depression (PROMIS-29 and PROPR dimension) |
| DCE | Discrete Choice Experiment |
| EQ-5D | EuroQol – Five Dimension |
| EQ-5D-3L | EuroQol – Five Dimension – Three Level |
| EQ-5D-5L | EuroQol – Five Dimension – Five Level |
| EQ-HWB | EuroQol – Health and Wellbeing measure |
| EQ-VT | EuroQol – Valuation Technology |
| FA | Fatigue (PROMIS-29 and PROPR dimension) |
| HRQoL | Health Related Quality of Life |
| IRT | Item Response Theory |
| MO | Mobility (EQ-5D dimension) |
| PA | Pain (PROMIS-29 and PROPR dimension) |
| PD | Pain and Discomfort (EQ-5D dimension) |
| PF | Physical Functioning (PROMIS-29 and PROPR dimension) |
| PROMIS | Patient Reported Outcome Measurement Information System |
| PROMIS-29 | Patient Reported Outcome Measurement Information System – 29 item fixed form |
| PROPR | PROMIS-Preference scoring system |
| QALY | Quality Adjusted Life Year |
| QoL | Quality of Life |
| SC | Self-Care (EQ-5D dimension) |
| SF-6D | Short Form – Six Dimension |
| SF-6Dv2 | Short Form – Six Dimension Version 2 |
| SF-12 | Short Form – 12 item questionnaire |
| SF-36 | Short Form – 36 item questionnaire |
| SL | Sleep (PROMIS-29 and PROPR dimension) |
| SOC | Social Limitations (PROMIS-29 and PROPR dimension) |
| TTO | Time Trade Off |
| UA | Usual Activities (EQ-5D dimension) |

**Appendix 2. Frequency of responses to each EQ-5D-5L dimension**

| **Dimension** | **No problems** | **Slight** | **Moderate** | **Severe** | **Extreme** |
| --- | --- | --- | --- | --- | --- |
| Mobility | 485 (61.1) | 186 (23.4) | 93 (11.7) | 25 (3.2) | 5 (0.6) |
| Self Care | 667 (84.0) | 72 (9.1) | 44 (5.5) | 6 (0.8) | 5 (0.6) |
| Usual Activities | 439 (55.3) | 224 (28.2) | 102 (12.9) | 24 (3.0) | 5 (0.6) |
| Pain/Discomfort | 238 (30.0) | 336 (42.3) | 151 (19.0) | 55 (6.9) | 14 (1.8) |
| Anxiety/Depression | 378 (47.6) | 216 (27.2) | 133 (16.8) | 47 (5.9) | 20 (2.5) |

**Appendix 3: Frequency of top 15 EQ-5D-5L health states and top 5 PROMIS-29 responses**

| **Rank** | **EQ-5D-5L State** | **N(%)** | **N of different PROMIS-29 states** |  |
| --- | --- | --- | --- | --- |
| 1 | 11111 | 139 (17.5) | 41,40.3,57,41.6,33.7,32,64.2 | 14 (1.8) |
| 2 | 11121 | 91 (11.46) | 41,40.3,57,41.6,33.7,37.5,64.2 | 8 (1.0) |
| 3 | 11122 | 60 (7.56) | 41,40.3,57,41.6,48.6,41.1,64.2 | 6 (0.8) |
| 4 | 11112 | 34 (4.28) | 41,40.3,57,41.6,48.6,46.2,64.2 | 6 (0.8) |
| 5 | 21221 | 23 (2.9) | 41,40.3,57,41.6,33.7,43.8,64.2 | 4 (0.5) |
| =6 | 11113 | 16 (2.0) |  |  |
| =6 | 11221 | 16 (2.0) |  |  |
| =8 | 21121 | 15 (1.9) |  |  |
| =8 | 21222 | 15 (1.9) |  |  |
| =10 | 11123 | 14 (1.8) |  |  |
| =10 | 11222 | 14 (1.8) |  |  |
| 12 | 21122 | 12 (1.5) |  |  |
| 13 | 11223 | 11 (1.4) |  |  |
| =14 | 21111 | 10 (1.3) |  |  |
| =14 | 21231 | 10 (1.3) |  |  |

EQ-5D-5L dimension order: MO, SC, UA, PD, AD; PROMIS-29 domain order: D, A, PF, PA, FA, SL, SOC; PROMIS coded by T-Score

**Appendix 4: Frequency of respondents at the ceiling and floor of each PROMIS-29 domain, the domain internal consistency**

| **Domain** | **Ceiling** | **Floor** |
| --- | --- | --- |
| Anxiety | 198 (24.9) | 7 (0.88) |
| Depression | 246 (30.1) | 14 (1.8) |
| Physical functioning | 293 (36.9) | 6 (0.8) |
| Pain | 264 (33.3) | 19 (2.4) |
| Fatigue | 75 (9.5) | 35 (4.4) |
| Sleep | 33 (4.2) | 21 (2.6) |
| Social Limitations | 168 (21.2) | 19 (2.4) |

**Appendix 5: EQ-5D-5L dimension correlations**

|  | Mobility | Self Care | Usual Activities | Pain/Discomfort |
| --- | --- | --- | --- | --- |
| Mobility | - | - | - | - |
| Self Care | **0.60** | - | - | - |
| Usual Activities | **0.70** | **0.57** | - | - |
| Pain/Discomfort | **0.60** | 0.39 | **0.61** | - |
| Anxiety/Depression | 0.22 | 0.23 | 0.42 | 0.32 |

**Appendix 6: PROMIS-29 dimension correlations**

|  | Depression | Anxiety | Physical Functioning | Pain | Fatigue | Sleep |
| --- | --- | --- | --- | --- | --- | --- |
| Depression | - | - | - | - | - | - |
| Anxiety | **0.84** | - | - | - | - | - |
| Physical Functioning | -0.37 | -0.40 | - | - | - | - |
| Pain | 0.46 | 0.47 | **0.65** | - | - | - |
| Fatigue | **0.60** | **0.63** | 0.42 | **0.50** | - | - |
| Sleep | **0.53** | **0.58** | 0.37 | 0.48 | **0.60** | - |
| Social Limitations | **-0.62** | **-0.64** | **-0.62** | **-0.71** | **-0.63** | **-0.58** |

**Appendix 7: Known group validity for top five reported conditions**

|  |  | **EQ-5D-5L Australian** | | | **EQ-5D-5L United States** | | | **PROPr** | | |
| --- | --- | --- | --- | --- | --- | --- | --- | --- | --- | --- |
| **Condition** | **N** | **Mean (SD)** | **ES (95% CI)** | **Sig** | **Mean (SD)** | **ES (95% CI)** | **Sig** | **Mean (SD)** | **ES (95% CI)** | **Sig** |
| Pain |  |  | **1.10 (0.92 – 1.24)** | <0.001 |  | **1.20 (1.01 – 1.34)** | <0.001 |  | **0.94 (0.78 – 1.10)** | <0.001 |
| No | 564 | 0.777 (0.218) |  |  | 0.836 (0.181) |  |  | 0.463 (0.225) |  |  |
| Yes | 228 | 0.511 (0.305) |  |  | 0.577 (0.297) |  |  | 0.262 (0.184) |  |  |
| Tiredness |  |  | 0.77 (0.61 – 0.93) | <0.001 |  | 0.69 (0.53 – 0.85) | <0.001 |  | **1.01 (0.85 – 1.17)** | <0.001 |
| No | 575 | 0.755 (0.254) |  |  | 0.806 (0.230) |  |  | 0.464 (0.224) |  |  |
| Yes | 217 | 0.556 (0.273) |  |  | 0.640 (0.261) |  |  | 0.250 (0.174) |  |  |
| Depression |  |  | **1.25 (1.07 – 1.42)** | <0.001 |  | **1.05 (0.88 – 1.21)** | <0.001 |  | **1.11 (0.94 – 1.28)** | <0.001 |
| No | 597 | 0.775 (0.224) |  |  | 0.820 (0.209) |  |  | 0.464 (0.222) |  |  |
| Yes | 195 | 0.473 (0.281) |  |  | 0.579 (0.277) |  |  | 0.229 (0.161) |  |  |
| Anxiety |  |  | **0.93 (0.75 – 1.10)** | <0.001 |  | **0.79 (0.60 – 0.95)** | <0.001 |  | **0.99 (0.80 – 1.15)** | <0.001 |
| Low | 623 | 0.752 (0.258) |  |  | 0.801 (0.235) |  |  | 0.451 (0.227) |  |  |
| High | 169 | 0.511 (0.249) |  |  | 0.614 (0.249) |  |  | 0.238 (0.164) |  |  |
| Hypertension |  |  | 0.47 (0.30 – 0.63) | <0.001 |  | 0.58 (0.41 – 0.75) | <0.001 |  | 0.36 (0.20 – 0.53) | <0.001 |
| No | 613 | 0.728 (0.258) |  |  | 0.789 (0.230) |  |  | 0.423 (0.233) |  |  |
| Yes | 181 | 0.600 (0.310) |  |  | 0.656 (0.294) |  |  | 0.338 (0.220) |  |  |
